# Machine learning-derived prediction of in-hospital mortality in patients with severe acute respiratory infection: analysis of claims data from the German-wide Helios hospital network

**DOI:** 10.1186/s12931-022-02180-w

**Published:** 2022-09-23

**Authors:** Johannes Leiner, Vincent Pellissier, Sebastian König, Sven Hohenstein, Laura Ueberham, Irit Nachtigall, Andreas Meier-Hellmann, Ralf Kuhlen, Gerhard Hindricks, Andreas Bollmann

**Affiliations:** 1 Department of Electrophysiology, Heart Center Leipzig at University of Leipzig, Leipzig, Germany; 2 Real World Evidence and Health Technology Assessment, Helios Health Institute, Berlin, Germany; 3 Helios Hospitals, Berlin, Germany; 4 Helios Health, Berlin, Germany; 5 Department of Infectious Diseases and Infection Prevention, Helios Hospital Emil-von-Behring, Berlin, Germany; 6 Institute of Hygiene and Environmental Medicine, Charité - Universitaetsmedizin Berlin, Berlin, Germany; 7 Clinic for Cardiology, University Hospital Leipzig, Leipzig, Germany

**Keywords:** Mortality prediction models, Machine learning, Severe acute respiratory infection, Administrative data

## Abstract

**Background:**

Severe acute respiratory infections (SARI) are the most common infectious causes of death. Previous work regarding mortality prediction models for SARI using machine learning (ML) algorithms that can be useful for both individual risk stratification and quality of care assessment is scarce. We aimed to develop reliable models for mortality prediction in SARI patients utilizing ML algorithms and compare its performances with a classic regression analysis approach.

**Methods:**

Administrative data (dataset randomly split 75%/25% for model training/testing) from years 2016–2019 of 86 German Helios hospitals was retrospectively analyzed. Inpatient SARI cases were defined by ICD-codes J09-J22. Three ML algorithms were evaluated and its performance compared to generalized linear models (GLM) by computing receiver operating characteristic area under the curve (AUC) and area under the precision-recall curve (AUPRC).

**Results:**

The dataset contained 241,988 inpatient SARI cases (75 years or older: 49%; male 56.2%). In-hospital mortality was 11.6%. AUC and AUPRC in the testing dataset were 0.83 and 0.372 for GLM, 0.831 and 0.384 for random forest (RF), 0.834 and 0.382 for single layer neural network (NNET) and 0.834 and 0.389 for extreme gradient boosting (XGBoost). Statistical comparison of ROC AUCs revealed a better performance of NNET and XGBoost as compared to GLM.

**Conclusion:**

ML algorithms for predicting in-hospital mortality were trained and tested on a large real-world administrative dataset of SARI patients and showed good discriminatory performances. Broad application of our models in clinical routine practice can contribute to patients’ risk assessment and quality management.

**Supplementary Information:**

The online version contains supplementary material available at 10.1186/s12931-022-02180-w.

## Introduction

Severe acute respiratory infection (SARI) has been defined by the World Health Organization (WHO) in 2011 and is described by the following criteria: acute respiratory illness, history of fever (or measured fever of ≥ 38 degrees Celsius) cough, dyspnea (or tachypnoea), onset within the past 10 days, required hospitalization [1, 2]. Several outbreaks of SARI in recent years were reported, mostly due to influenza viruses [3, 4]. According to the Global Burden of Diseases Study 2015, lower respiratory tract infections are the most common infectious causes of death [5]. Not only since the onset of the global SARS-CoV2 pandemic in 2019 the importance of epidemiological research on SARI-related hospital admissions was acknowledged. Large-scale prospective studies and hospital-based surveillance systems were established in the last decade as a response to past epidemics [1, 6, 7] including the German ICOSARI-sentinel, an ongoing SARI surveillance system conducted by the German federal government agency Robert-Koch-Institute (RKI) in collaboration with Helios Kliniken GmbH [8].

The capability of machine learning (ML) algorithms to predict patient outcomes has been studied among different disease entities [9]. For example, the outcome prediction in COVID-19 patients using deep learning methods was recently evaluated with promising results [10–12]. With respect to non-COVID SARI patients, several approaches for mortality prediction in patients with pneumonia in general and specifically with influenza-caused pneumonia were also reported. The methodology of those studies included different ML concepts [13–16] as well as logistic regression (LR) [14, 17, 18]. The authors mostly focused on developing individual risk stratification and mortality prediction models for assessing the patient’s individual risk at the time point of hospital admission. Pointing into the same direction, several well-established assessment tools exist for pneumonia to evaluate individual mortality risk and help guiding the clinicians’ decisions. Widely used scores are the CRB-65/CURB-65 [19, 20] and Pneumonia Severity Index (PSI) [21, 22]. However, predicting outcomes also on a population rather than an individual level is necessitated in context of public health interests and research as well as hospital benchmarking. Prediction tools with this purpose are lacking.

In regard of cardiovascular diseases, several studies with focus on risk stratification have been performed [23, 24] also by applicating ML approaches [25]. Our working group recently presented an analysis on in-hospital mortality in heart failure (HF) patients with implementation of ML algorithms [26]. This preliminary work on population-based risk prediction has provided us with an established methodological concept that forms the basis for this study in the scope of SARI. In a more patient-based approach, we aimed to evaluate mortality prediction models for SARI patients and in this context compare different ML algorithms with LR (generalized linear models, GLM).

## Methods

### Case definition

Different case definitions exist to identify SARI patients from administrative data in a hospital setting considering that not all SARI-defining conditions can be assessed by this data source. In the above mentioned ICOSARI-sentinel, one approach used SARI-specific main and secondary diagnoses of ICD-10-codes (International Statistical Classification of Diseases and Related Health Problems Version 10) J09-J22 for case definition and proved to be sensitive [8]. This method was adapted in our study. ICD-10-codes J09-J22 comprise influenza and pneumonia (J09-J18), acute bronchitis (J20.-), acute bronchiolitis (J21.-) and unspecified acute lower respiratory tract infection (J22) [27].

### Data source

Our dataset included administrative data from 86 hospitals within the German Helios network. Inclusion criteria were (1) inpatient treatment and (2) SARI as main or secondary diagnosis as defined by ICD-10-codes (see above). We retrospectively analyzed urgent or regular patient admissions from January 1^st^ 2016 to December 31^st^ 2019. In-hospital death as the primary outcome measure of interest was identified via the type of discharge. ICD-10-GM-codes (German Modification of the ICD-10) as main and secondary diagnoses at hospital discharge were used to identify relevant comorbidities according to the Elixhauser comorbidity score without distinguishing between preexisting comorbidities and newly diagnosed conditions [28, 29]. A detailed overview of ICD-10-GM-codes and the Elixhauser comorbidity score [29] is provided in the (Additional file 1: Table S1). The analysis was carried out according to the principles outlined in the Declaration of Helsinki. Patient-related data were stored in a anonymized form. The local ethics committee (vote: AZ490/20-ek) and the Helios Kliniken GmbH data protection authority approved data use for this study.

### Statistical analysis

The methodological approach presented here was successfully applied to a dataset of HF patients before and was used similarly for this analysis [26]. The initial dataset was split randomly into 75% used for model development (model training) and 25% for model testing. The dataset splits were performed so that all the cases for a given patient were in the same subset (train/tests or train/validation for cross-validation approach). The outcome probability was identical in each subset. Each variable set contained the following baseline variables: age, gender, admission year, ICU treatment (yes/no), hospital-acquired SARI (yes/no) and SARI type. For the latter, we subdivided the ICD-codes for SARI (J09-J22) to define different SARI types: influenza J09, J10; viral pneumonia other than influenza J12; bacterial pneumonia J13-J16; other pneumonia J17, J18; other lower respiratory tract infections J20-J22.

In a first step, we evaluated and cross-validated two different variable sets based on the training dataset: one contained Elixhauser comorbidities as separate variables and one contained the Elixhauser weighted comorbidity scores [29].

Variables which were highly sparse and unbalanced (near-zero variance variables [26]), were removed prior to the analysis. No variables were highly correlated. This concerned several Elixhauser comorbidities. Additionally, the SARI types “influenza” and “viral pneumonia other than influenza” were removed prior to model training because of the low case numbers (4.2% and 1.5% respectively, see Additional file 1: Table S2). All continuous variables were scaled and centered before the analyses. The dataset did not contain any missing values.

The two variable sets were evaluated using four different algorithms applied on the training dataset: GLM, random forest (RF), single layer neural network (NNET) and extreme gradient boosting (XGBoost).

Model tuning was carried out in accordance to previous descriptions [26] using a Bayesian model based optimization method with a k-folds approach using one repetition of 10-folds each. While ML approaches can implicitly account for non-linearities, these have to be explicit in GLM. Non-linearities were accounted for using a polynomial on continuous variables (age and Elixhauser score) and the number of degrees was tuned using the method described above. To evaluate the performance of the models trained, the values predicted during the cross-validation process were used to compute receiver operating characteristic (ROC) area under the curve (AUC) and area under the precision-recall curve (AUPRC). The model with the highest AUPRC was considered the best. To assess the relative importance of the variables used, we performed a Shapley Additive exPlanations (SHAP) analysis separately for each algorithm, which is an approach to explain variable importance that is agnostic to the type of model and therefore facilitates a comparison [30]. The predictive abilities of each algorithm were assessed with the ROC curve, the precision-recall curve, calibration-in-the-large, weak calibration and calibration plots, AUC and AUPRC. Calibration-in-the-large is simply a comparison of the observed vs. predicted risk, while weak calibration is the intercept and slope of the logistic regression between observed and predicted death [31]. DeLong’s test was used to perform pairwise comparisons between ROC AUCs [32]. All analyses were carried out within the R environment for statistical computing (Version 3.6.1, 64-bit built).

## Results

The final dataset included 241,988 SARI cases from 86 Helios hospitals. Baseline characteristics are summarized in the (Additional file 1: Table S2). Age and sex distribution showed that 49% of the patients were 75 years or older and 56.2% were male. 20% of the SARI cases were hospital-acquired and intensive care unit (ICU) treatment was required in 14.7% of patients. Regarding SARI type, numbers of influenza (4.2%) and viral pneumonia other than influenza (1.5%) were low and “other pneumonia” (J17, J18) was the most frequently observed SARI type (56.6%). In-hospital mortality rate was 11.6% overall and 31.6% in patients requiring an ICU therapy. Univariate regression analyses revealed advanced age, ICU treatment, hospital-acquired SARI, bacterial pneumonia and several Elixhauser comorbidities (e.g., congestive HF) as the strongest predictors of in-hospital mortality (Table 1). The cohort for model training and testing comprised 181,574 and 60,414 patients, respectively. Baseline characteristics were well balanced between groups with respect to all variables (Additional file 1: Table S2).

**Table 1:** Univariate regression analyses, predictors of in-hospital mortality

Variable	In-hospital mortality, n (% of patients with the same variable expression)	Odds Ratio (95% CI)	P-value
N (total)	28,025 (11.6)		
Age			
< 65	3835 (4.5)		
65–74	5,025 (12.8)	3.086 (2.953–3.224)	< 0.001
≥ 75	19,165 (16.2)	4.047 (3.904–4.195)	< 0.001
Gender			
Female	11,317 (10.7)		
Male	16,708 (12.3)	1.172 (1.143–1.202)	< 0.001
ICU treatment			
No	16,789 (8.1)		
Yes	11,236 (31.6)	5.206 (5.065–5.35)	< 0.001
Hospital-acquired SARI			
No	15,922 (8.2)		
Yes	12,103 (25)	3.712 (3.616–3.81)	< 0.001
Influenza			
No	27,514 (11.9)		
Yes	511 (5)	0.39 (0.356–0.427)	< 0.001
Viral pneumonia other than influenza			
No	27,957 (11.7)		
Yes	68 (1.9)	0.144 (0.113–0.183)	< 0.001
Bacterial pneumonia			
No	20,658 (10.1)		
Yes	7367 (19.5)	2.158 (2.096–2.222)	< 0.001
Other pneumonia			
No	8,700 (8.3)		
Yes	19,325 (14.1)	1.818 (1.77–1.867)	< 0.001
Other lower respiratory tract infections			
No	26,163 (14.3)		
Yes	1862 (3.2)	0.196 (0.187–0.206)	< 0.001
Congestive heart failure			
No	13,466 (8.4)		
Yes	14,559(17.8)	2.355 (2.296–2.415)	< 0.001
Cardiac arrhythmias			
No	15,068 (9)		
Yes	12,957 (17.2)	2.1 (2.047–2.153)	< 0.001
Valvular disease			
No	23,300 (10.9)		
Yes	4725 (16.6)	1.621 (1.567–1.677)	< 0.001
Pulmonary circulation disorders			
No	24,752 (11.1)		
Yes	3273 (16.7)	1.595 (1.533–1.66)	< 0.001
Peripheral vascular disorders			
No	23,501 (10.8)		
Yes	4524 (18.9)	1.922 (1.856–1.991)	< 0.001
Hypertension, uncomplicated			
No	17,555 (11.3)		
Yes	10,470 (12.1)	1.076 (1.049–1.104)	< 0.001
Hypertension, complicated			
No	22,929 (11.3)		
Yes	5096 (13.1)	1.186 (1.148–1.225)	< 0.001
Paralysis			
No	25,057 (11.1)		
Yes	2968 (18.9)	1.871 (1.794–1.951)	< 0.001
Other neurological disorders			
No	23,197 (10.7)		
Yes	4828 (18.6)	1.905 (1.841–1.971)	< 0.001
Chronic pulmonary disease			
No	22,520 (11.5)		
Yes	5505 (12)	1.051 (1.018–1.084)	0.002
Diabetes, uncomplicated			
No	23,342 (11.1)		
Yes	4683 (14.4)	1.343 (1.298–1.389)	< 0.001
Diabetes, complicated			
No	23,436 (11)		
Yes	4589 (15.9)	1.527 (1.476–1.581)	< 0.001
Hypothyroidism			
No	25,383 (11.6)		
Yes	2642 (11.2)	0.956 (0.917–0.998)	0.041
Renal failure			
No	14,923 (9.5)		
Yes	13,102 (15.6)	1.764 (1.72–1.808)	< 0.001
Liver disease			
No	25,094 (10.9)		
Yes	2931 (23.7)	2.525 (2.418–2.637)	< 0.001
Metastatic cancer			
No	24,609 (10.7)		
Yes	3416 (27.4)	3.147 (3.019–3.281)	< 0.001
Solid tumor without metastasis			
No	22,839 (10.4)		
Yes	5,186 (23.5)	2.653 (2.564–2.744)	< 0.001
Coagulopathy			
No	23,269 (10.2)		
Yes	4756 (32.4)	4.21 (4.056–4.369)	< 0.001
Obesity			
No	25,187 (11.7)		
Yes	2838 (10.5)	0.879 (0.844–0.916)	< 0.001
Weight loss			
No	20,416 (9.8)		
Yes	7609 (22.4)	2.655 (2.578–2.734)	< 0.001
Fluid and electrolyte disorders			
No	10,342 (7.5)		
Yes	17,683 (16.9)	2.51 (2.446–2.575)	< 0.001
Depression			
No	26,686 (11.7)		
Yes	1339 (10.1)	0.854 (0.806–0.905)	< 0.001

*ICU* Intensive care unit, *SARI* severe acute respiratory infection

### Model training

During the training process, the hyper-parameters of each algorithm (except for GLM, where only the number of degrees in polynomial was tuned) were tuned keeping the following values (two values were specified for variable sets containing either the Elixhauser comorbidities or the Elixhauser weighted comorbidity scores):

- GLM: number of degrees in polynomial age = 3/1, Elixhauser score = 1/na
- RF: number of variables randomly selected at each split = 4/3, number of trees = 1062/1168, minimum number of observations in each node = 39/32
- NNET: number of units in the hidden layer = 6/1, learning rate = 0.96/9e-6
- XGBoost: maximum number of boosting iterations = 2487/2926, maximum depth = 11/14; learning rate = 0.003/7e-5, minimum loss reduction = 0.001/0.0001; proportion of columns sampled per tree = 1; minimum child weight = 37/17; proportion of rows sampled per tree = 0.76/0.56

The cross-validation during model training showed a slightly better performance of the ML models when compared to GLM (AUC = 0.825; AUPRC = 0.365). The best-performing algorithm was XGBoost (AUC = 0.832; AUPRC = 0.388). The models containing separate Elixhauser comorbidities turned out to be superior to the Elixhauser score model among all algorithms used and were therefore kept during model testing. Plots of the SHAP analysis depicting variable importance for each algorithm are available from the Additional file 1: Fig. S1.

### Model testing

**Fig. 1:**
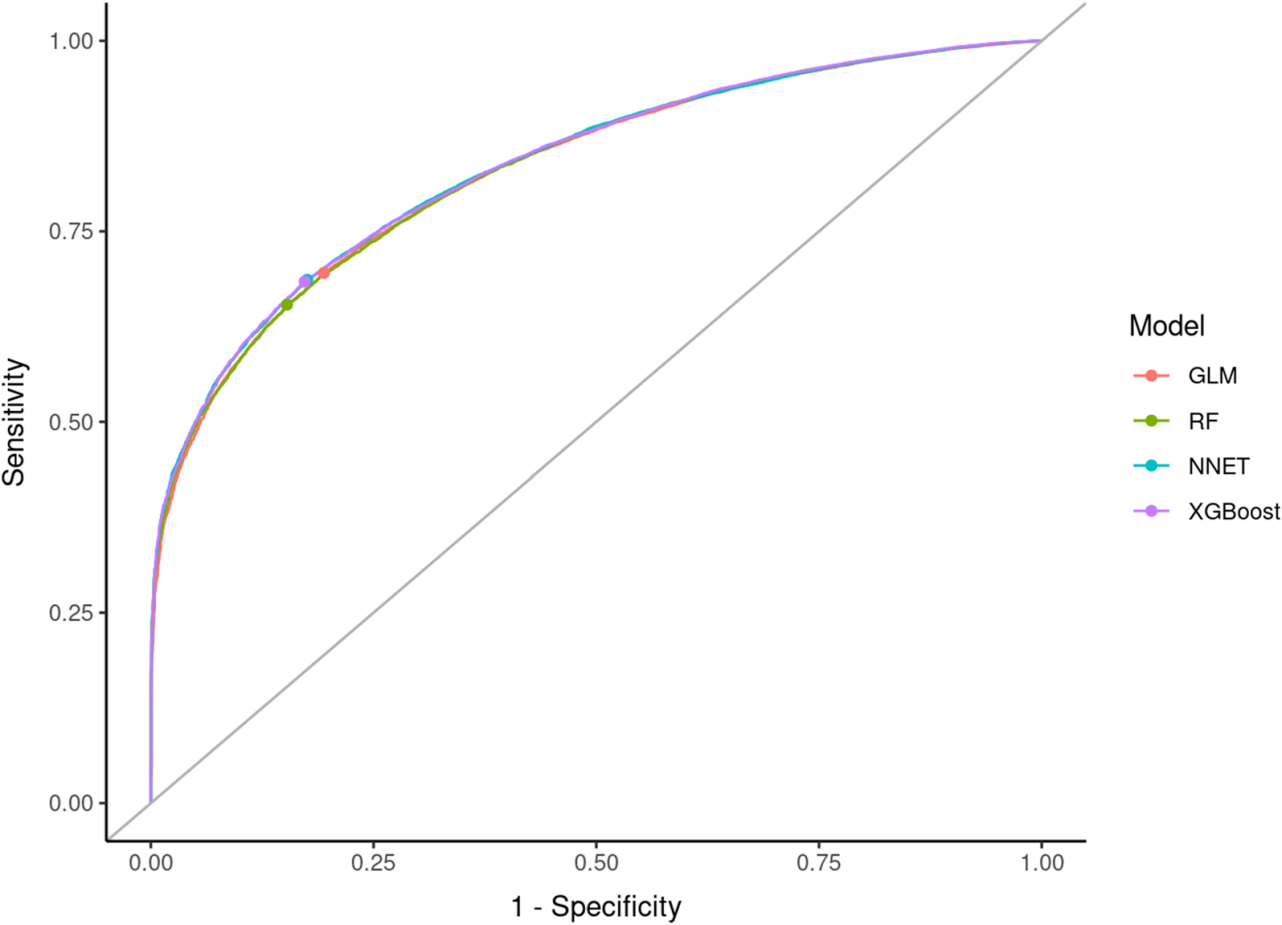
Receiver operating characteristic (ROC) curves (model testing). *GLM* generalized linear models, *NNET* single layer neural network, *RF* random forest, *XGBoost* extreme gradient boosting

**Fig. 2:**
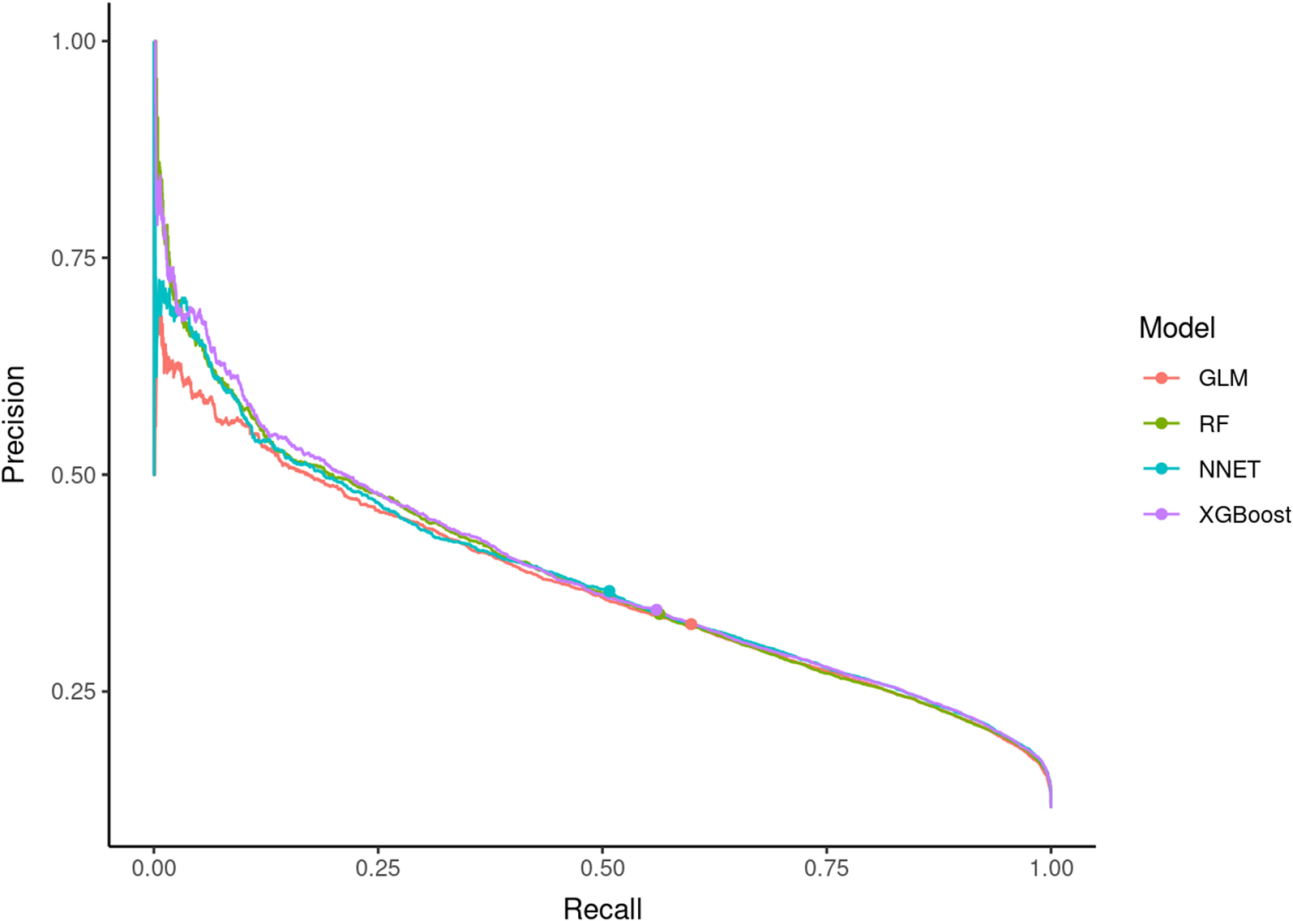
Precision-recall curves (model testing). *GLM* generalized linear models, *NNET* single layer neural network, *RF* random forest, *XGBoost* extreme gradient boosting

**Fig. 3:**
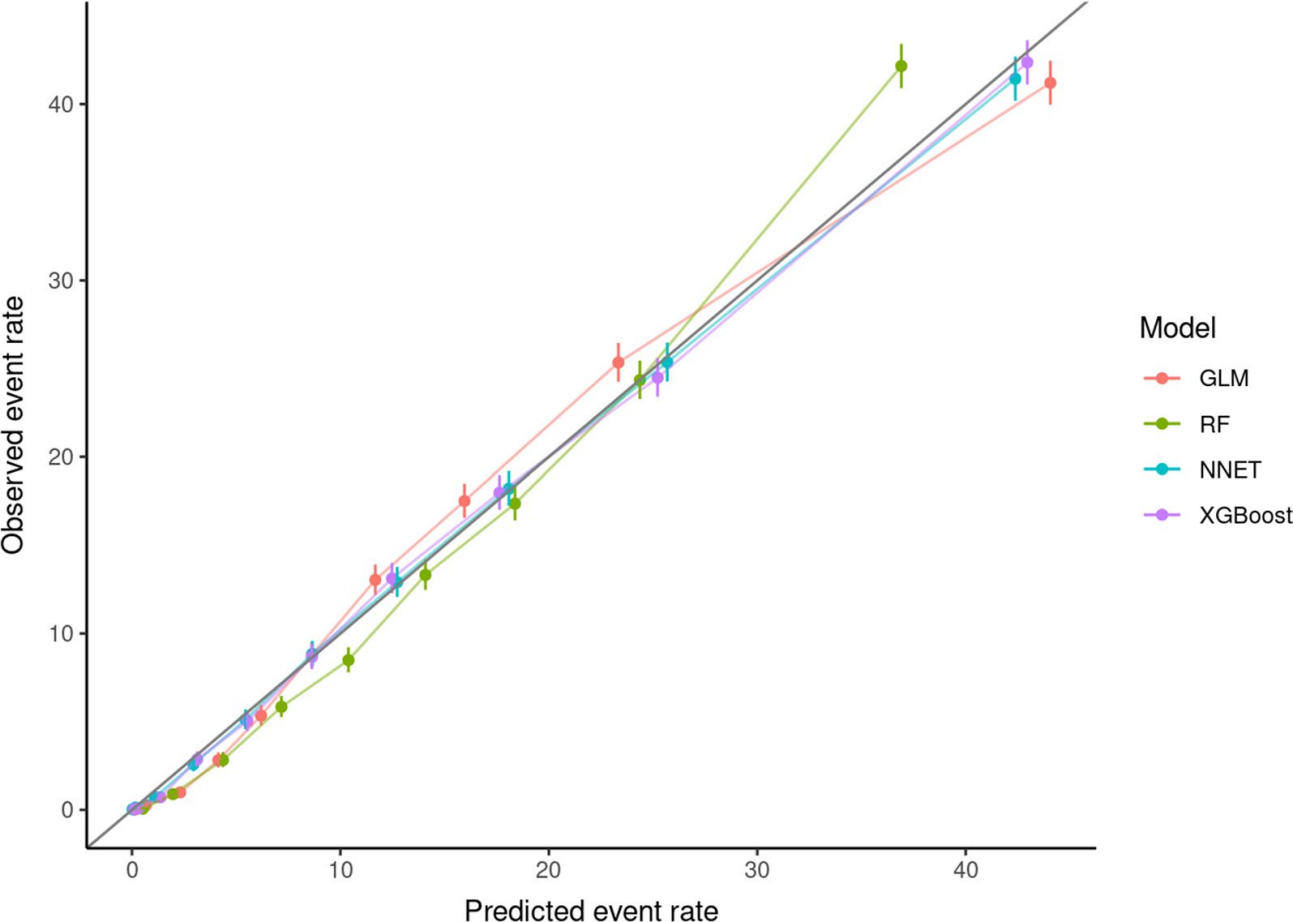
Calibration plots during model testing. *GLM* generalized linear models, *NNET* single layer neural network, *RF* random forest, *XGBoost* extreme gradient boosting. The straight bold line at 45 degrees illustrates perfect calibration

Applied to the testing cohort, the ML models did not markedly outperform GLM. Yet, a marginal better performance could be demonstrated for all three ML models, but confidence intervals (CIs) overlapped with those of GLM. AUCs and corresponding AUPRC with 95%CIs are given in Table 2. DeLong’s test[32] used for comparing ROC AUCs showed a significantly better performance of NNET and XGBoost in comparison to GLM (p < 0.001, Additional file 1: Table S3). Figures 1 and 2 show the ROC curves and corresponding precision-recall curves. Calibration metrics and calibration plots are shown in Table 3 and Fig. 3, respectively. The best calibration was observed with NNET and XGBoost models, followed by GLM, while RF displayed the worst calibration (over- as well as underestimation of mortality risk). Further performance metrics of all models can be found in the Additional file 1: Table S4.

**Table 2:** Model testing (Elixhauser comorbidities model)

Algorithm	AUC (95%CI)	AUPRC (95%CI)
GLM	0.83 (0.825–0.834)	0.372 (0.361–0.384)
RF	0.831 (0.827–0.835)	0.384 (0.373–0.396)
NNET	0.834 (0.83–0.838)	0.382 (0.371–0.393)
XGBoost	0.834 (0.83–0.839)	0.389 (0.378–0.4)

*95% CI* 95% confidence interval, *AUC* Area under the curve, *AUPRC* Area under the precision-recall curve, *GLM* generalized linear models, *NNET* single layer neural network, *RF* random forest, *XGBoost* extreme gradient boosting

**Table 3:** Calibration metrics

	Calibration-in-the-large	Calibration intercept (95%CI)	Calibration slope (95%CI)
GLM	11.5% (6969/60414) vs. 11.7%	− 0.02 (− 0.049 to 0.006)	1.02 (0.997 to 1.05)
RF	11.5% (6969/60414) vs. 11.8%	− 0.03 (− 0.059 to − 0.005)	1.26 (1.228 to 1.299)
NNET	11.5% (6969/60414) vs. 11.7%	− 0.02 (− 0.05 to 0.005)	1.01 (0.982 to 1.038)
XGBoost	11.5% (6969/60414) vs. 11.7%	− 0.02 (− 0.051 to 0.004)	1.03 (1.003 to 1.057)

*95% CI* 95% confidence interval, *GLM* generalized linear models, *NNET* single layer neural network, *RF* random forest, *XGBoost* extreme gradient boosting

## Discussion

In this study, we present real-world administrative data on in-hospital mortality of 241,988 patients with SARI which is derived from a nationwide German hospital network. Different ML mortality prediction models displayed an overall good discriminatory performance with respect to AUC and AUPRC. Compared to standard statistical methods (GLM), NNET and XGBoost showed a small but statistically significant difference in ROC AUCs. However, the relevance of this marginal better performance remains unknown from a clinical perspective and warrants further evaluation. Future studies are therefore needed to explore the usefulness and advantages of ML concepts in the context of outcome prediction.

The results highlight comorbidities as important influencing factors with respect to SARI-related deaths. Implementation of our mortality prediction models, utilizing only easily and widely available variables, in clinical care can help assessing the patients’ individual mortality risks and could moreover be useful for hospital benchmarking. We chose to include patient data from 2016 to 2019 for our analysis as the COVID-19 pandemic could have been a major influencing factor with regard to SARI mortality in 2020. This assumption should be investigated in future analyses also relating to a possible scalability of our proposed mortality prediction models in view of the ongoing pandemic.

### Mortality and clinical characteristics

Mortality data for SARI can be derived from large-scale prospective studies. In the globally conducted SPRINT-SARI trial, overall mortality rate is given as 9.5% and in patients > 60 years of age as 18.6% which is comparable to our findings (overall in-hospital mortality 11.6%) and may be an indicator of the good reliability of our retrospective claims-based dataset. Organ dysfunction as assessed by SOFA-scores (sequential organ failure assessment) at initial patient presentation and increased age were identified as independent predictors of in-hospital mortality in this study [1]. Higher mortality rates (ICU mortality: 20.2%; in-hospital mortality: 27.2%) among ICU-admitted patients with SARI are reported in the IC-GLOSSARI trial [7]. The higher ICU mortality that was seen in our study (20.2 vs. 31.6%, Table 1) could be attributed to a different risk profile in regard of cardiovascular and non-cardiovascular comorbidities (e.g., congestive heart failure, cardiac arrhythmias, renal failure) which were less frequently observed among patients in the IC-GLOSSARI trial.

In a recent analysis of the ICD-code based ICOSARI-sentinel [8], 5-year data from German hospitals of influenza waves (2015–2019; week numbers 3–11) were compared to outcomes of COVID-19 patients. Analyses of almost 70.000 patients admitted with SARI showed an overall mortality rate of 12%, ICU admissions in 32% of the cases and an ICU mortality rate of 22% [33]. The overall mortality rate that was observed in this analysis is almost similar to our data (in-hospital mortality 11.6%) while there were fewer ICU admissions (14.7%) and a higher ICU mortality rate (31.6%). The observed differences may be due to the selective choice of data from influenza wave periods in the ICOSARI-sentinel while our dataset included the whole year periods 2016–2019. Furthermore, diverging ICU admission rates could have been caused by varying definitions of ICU treatment and respective monitoring when using an administrative data source.

In another presentation of ICOSARI-data, the investigators reported unexpectedly low numbers of influenza (defined by ICD-codes) among SARI cases in general which is in accordance to our findings (only 4.2% of the cases accounted for influenza, Additional file 1: Table S2) [8].

Of note, univariate regression analyses (Table 1) revealed obesity as a rather protective factor regarding in-hospital mortality. This finding contrasts with recent experiences during the COVID-19 pandemic where obese patients display a greater risk for mortality [34]. However, it has been shown that obesity is paradoxically associated with lower mortality rates among ICU patients [35] and patients with ARDS [36] which may be explanatory for our observation.

### Existing prediction models and comparison

The use of administrative data and its validity for assessing and predicting in-hospital mortality has been studied thoroughly in patients with cardiovascular diseases [26, 37–40] but previous work on respiratory tract infections in that matter is scarce. One US-study compared administrative data and electronic medical records (EMR) as data sources for developing a model to calculate hospital-specific risk-standardized 30-day mortality rates in patients with pneumonia [17]. An important finding was the good agreement between mortality estimates derived from administrative data and EMR respectively, which underlines the usefulness and reliability of claims data sets to assess clinical outcomes. However, Bratzler et al. used GLM only in their study on 224,608 pneumonia patients and the administrative data model provided an AUC of 0.72 which is considerably lower than the presented AUCs of our ML models and GLM [17]. The comorbidity variables that were included in the model by Bratzler et al. were comparable to the Elixhauser comorbidities but only age and gender were used as administrative variables in contrast to our approach where we also took for example ICU treatment and whether the SARI was hospital-acquired into account.

A Japanese working group analyzed a claims data set with 35,297 patients hospitalized for community acquired pneumonia (CAP) comparing different models with the A-DROP score, a modified version of the CURB-65 score [41], by adding and excluding specific clinical variables and applying hierarchical LR[18]. The authors pursued the objective to develop risk-adjusted prediction models to facilitate hospital benchmarking. The newly developed models performed equally or better when compared to the A-DROP score with considerably higher AUC when compared to our results in range of 0.852–0.874 [18]. However, the authors utilized clinical variables, which are very specific for CAP (e.g., presence of infiltrations on chest x-ray) or may not be available and gathered on a routine basis (e.g., specific laboratory values) which hinders scalability and may impede implementation in certain hospitals or patient cohorts due to modest data availability on a population level and in routine care.

With regard to ML application for outcome prediction in patients with respiratory diseases, Hu et al. presented a retrospective study on 336 cases with severe influenza. XGBoost and RF algorithms provided an AUC of 0.842 and 0.809 in predicting 30-day mortality and outperformed LR and certain clinical prognostic scores (PSI, APACHE II) which highlights the usefulness of ML for outcome assessment also in critically ill patients [15]. From our perspective, limitations especially regarding applicability in the study by Hu et al. arise in view of the small case number and choice of a large variable set (76 variables). In a recently published US study, ML algorithms were applied using PSI-specific and additional variables derived from electronic health records (EHR) of 297,498 CAP patients [14]. The ML methods outperformed LR among different models in predicting 30-day mortality (AUC range 0.83–0.87). These results compare well with our observations on the discriminatory performance of ML approaches whereas significant superiority to GLM could not be demonstrated. This may indicate a good consistency between administrative and EHR datasets albeit different patient populations can only be compared with each other to a limited extent.

Another interesting approach to predict patient-specific mortality in CAP was reported by Wu et al. It comprised disjunctive normal forms learning algorithms which were compared to ML with promising results [16]. However, comparability to this study is very limited as specific cytokines, cell surface markers and single nucleotide polymorphisms were used as underlying variables for the models.

We assessed the predictive abilities of our algorithms not only with ROC AUC but also with AUPRC and calibration plots. The two best performing algorithms (XGBoost and NNET) also showed very good calibration (Fig. 3, Table 3). When evaluating models trained on datasets with a high outcome imbalance, precision-recall curves are often preferred over ROC curves [42]. In our case, we observed 11.6% in-hospital mortality and therefore a relatively low rate of true positives. Hence, the AUPRC is an important metric for performance evaluation of our ML models. When interpreting AUPRC values, the true positive rate in the dataset has to be considered, meaning that a value of 0.389 (XGBoost, Table 2) suggests good discrimination. However, none of the above-discussed studies presented precision-recall curves, so models had to be compared by means of ROC AUC as the most frequently utilized metric.

### Clinical risk scores

As mentioned before, several well-established risk scores for SARI patients exist which represent important clinical tools and can help treating physicians to assess SARI severity and the individual mortality risk at the time of the patient’s hospital admission, for example in an emergency room setting. The more complex PSI which comprises comorbidities, clinical parameters and results from laboratory analyses and instrumental examinations tends to provide better accuracy in predicting 30-day mortality when compared to CURB-65 [22] and A-DROP [41] with respective AUCs for PSI in the range of 0.72–0.89 [22].

### Clinical application

Our proposed mortality prediction models should be broadly applicable in clinical routine practice as administrative data is commonly available in hospital information systems (HIS). Automatic data extraction and implementation of risk score calculators in the HIS is conceivable. Individual risk prediction at the time point of the patients’ hospital admission or after a SARI diagnosis is established during a hospital stay could assist the physician in estimating disease severity. For CAP, it has been shown that this initial assessment of disease severity is crucial [43]. Differentiation between high risk and low risk patients would ultimately improve clinical decision-making and the quality of patient care.

In a population-based approach, these models can furthermore be used to calculate standardized mortality ratios for different patient cohorts, differentiated for instance according to specific geographic regions, time periods and hospitals and can hence serve as a basis for quality of care evaluation and assurance. However, external validation of our models among different patient cohorts is required to prove applicability and its benefits.

### Limitations

We acknowledge several limitations in connection with this study. First, we used retrospectively collected data only which is widely seen as of inferior quality in comparison to prospective studies. However, as has been shown above, mortality rates in our dataset did not differ markedly when compared to prospective studies. Second, some limitations must be attributed to claims-based datasets in general, as the collected data is not stored for research purposes but for administrative and remuneration reasons. The validity of the datasets is dependent on correct coding and cannot always be ensured if no control variables exist (e.g., medical records) as has been stated before [44]. However, the above mentioned work by Bratzler et al. [17] showed good correlation of claims data with EMR in pneumonia patients. Additionally, we must acknowledge that this kind of correlation and validation analysis by using EMR was not performed in our study. Third, we acknowledge that no validation to an external dataset took place. However, the dataset was derived from a network consisting of 86 hospitals in different German areas and therefore reflects well the nationwide state of patient care in context of SARI. Fourth, inclusion of more specific variables like laboratory values etc. could have improved the model accuracy but as our aim was to develop easy to apply models this was not found necessary.

## Conclusion

Our results show that the application of ML algorithms together with the use of routinely available administrative data is feasible for mortality prediction in SARI patients. In a large real-world multicenter cohort, ML approaches performed slightly better when compared to regression analysis. Implementation of our models into a clinical or quality management context could contribute decisively to risk stratification and hospital benchmarking respectively and ultimately could improve the quality of patient care.

## Supplementary Information

**Additional file 1: Table S1.** ICD-10-GM-codes used to calculate Elixhauser comorbidity score (according to Moore et al. [29]). **Table S2.** Baseline characteristics total dataset, training and testing cohort. **Table S3.** DeLong’s test for pairwise comparison of ROC AUCs. **Table S4.** Performance metrics (model testing). **Figure S1.** SHAP (SHapley Additive exPlanations) analysis for variable importance

## Data Availability

The data that support the findings of this study are not publicly available as they contain information that could compromise the privacy of research participants but are available from the corresponding author, Mr. Johannes Leiner (Johannes.leiner@helios-gesundheit.de) upon reasonable request. Same applies for the code used in development of our machine learning models.

## Abbreviations

A-DROP: Age, dehydration, respiratory failure, orientation disturbance, blood pressure
APACHE II: Acute physiology and chronic health evaluation score II
AUC: Receiver operating characteristic area under the curve
AUPRC: Area under the precision-recall curve
CAP: Community acquired pneumonia
CI: Confidence interval
COVID-19: Coronavirus disease 2019
CURB-65: Confusion, urea, respiratory rate, blood pressure, age ≥ 65
EHR: Electronic health records
EMR: Electronic medical records
GLM: Generalized linear models
HF: Heart failure
HIS: Hospital information system
HSMR: Hospital standardized mortality ratio
ICD: International Statistical Classification of Diseases
IC-GLOSSARI: Intensive Care GLObal Study on Severe Acute Respiratory Infection
ICOSARI: ICD-10-code based SARI-surveillance in Germany
NNET: Single layer neural network
PSI: Pneumonia Severity Index
RF: Random forest
RKI: Robert-Koch-Institute
ROC: Receiver operating characteristic
SARI: Severe acute respiratory infection
SARS-CoV2: Severe acute respiratory syndrome coronavirus 2
SHAP: Shapley Additive exPlanations
SOFA: Sequential organ failure assessment
SPRINT-SARI: Short PeRiod IncideNce sTudy of Severe Acute Respiratory Infection
WHO: World Health Organization
XGBoost: Extreme gradient boosting
