# Supplementary material for: Machine learning-derived prediction of in-hospital mortality in patients with severe acute respiratory infection: analysis of claims data from the German-wide Helios hospital network
Source: Respir Res. 2022 Sep 23;23:264. doi: 10.1186/s12931-022-02180-w (PMC9502925; doi:10.1186/s12931-022-02180-w)
Supplement: Supplementary file 1 — Additional file 1: Table S1. ICD-10-GM-codes used to calculate Elixhauser comorbidity score (according to Moore et al. [29]). Table S2. Baseline characteristics total dataset, training and testing cohort. Table S3. DeLong’s test for pairwise comparison of ROC AUCs. Table S4. Performance metrics (model testing). Figure S1. SHAP (SHapley Additive exPlanations) analysis for variable importance [file 12931_2022_2180_MOESM1_ESM.docx]

Machine Learning-derived prediction of in-hospital mortality

in patients with Severe Acute Respiratory Infection:

Analysis of claims data from the German-wide Helios Hospital Network

Johannes Leiner^1,2*^, MD; Vincent Pellissier^1,2*^, PhD; Sebastian König^1,2^, MD;

Sven Hohenstein^1,2^, PhD; Laura Ueberham^1,2^, MD; Irit Nachtigall^3^, MD;

Andreas Meier-Hellmann^3^, MD; Ralf Kuhlen^4^, MD, PhD;

Gerhard Hindricks^1,2^, MD and Andreas Bollmann^1,2^, MD, PhD

*first two authors contributed equally to this manuscript

Additional file 1

**Table S1: ICD-10-GM-codes used to calculate Elixhauser comorbidity score (according to Moore et al., 2017)**

| Elixhauser comorbidity | Weight | ICD-10-GM-code |
| --- | --- | --- |
| AIDS / HIV | 0 | B20, B21, B22, B23, B24 |
| Alcohol Abuse | -1 | F10, E52, G62.1, I42.6, K29.2, K70.0, K70.3, K70.9, T51, Z50.2, Z71.4, Z72.1 |
| Blood Loss Anemia | -3 | D50.0 |
| Cardiac Arrhythmia | 0 | I44.1, I44.2, I44.3, I45.6, I47, I48, I49, R00.0, R00.1, R00.8, T82.1, Z45.00, Z45.01, Z95.0 |
| Chronic Pulmonary Disease | 3 | I27.8, I27.9, J40, J41, J42, J43, J44, J45, J46, J47, J60, J61, J62, J63, J64, J65, J66, J67, J68.4, J70.1, J70.3 |
| Chronic Renal Failure | 6 | I12.0, I31.1, N18, N19, N25.0, Z49.0, Z49.1, Z49.2, Z94.0, Z99.2 |
| Coagulopathy | 11 | D65, D66, D67, D68, D69.1, D69.3, D69.4, D69.5, D69.6 |
| Congestive Heart Failure | 9 | I09.0, I11.0, I13.0, I13.2, I25.5, I42.0, I42.1, I42.2, I42.5, I42.6, I42.7, I42.8, I42.9, I43, I50 |
| Deficiency Anemia | -2 | D50.8, D50.9, D51, D52, D53 |
| Depression | -5 | F20.4, F31.3 - F31.5, F32, F33, F34.1, F41.2, F43.2 |
| Diabetes Mellitus, Uncomplicated | 0 | E10.0, E10.1, E10.9, E11.0, E11.1, E11.9, E12.0, E12.1, E12.9, E13.0, E13.1, E13.9, E14.0, E14.1, E14.9 (excluding E10.2, E10.3, E10.4, E10.5, E10.6, E10.7, E10.8, E11.2, E11.3, E11.4, E11.5, E11.6, E11.7, E11.8, E12.2, E12.3, E12.4, E12.5, E12.6, E12.7, E12.8, E13.2, E13.3, E13.4, E13.5, E13.6, E13.7, E13.8, E14.2, E14.3, E14.4, E14.5, E14.6, E14.7, E14.8) |
| Diabetes Mellitus, Complicated | -3 | E10.2, E10.3, E10.4, E10.5, E10.6, E10.7, E10.8, E11.2, E11.3, E11.4, E11.5, E11.6, E11.7, E11.8, E12.2, E12.3, E12.4, E12.5, E12.6, E12.7, E12.8, E13.2, E13.3, E13.4, E13.5, E13.6, E13.7, E13.8, E14.2, E14.3, E14.4, E14.5, E14.6, E14.7, E14.8 |
| Drug Abuse | -7 | F11, F12, F13, F14, F15, F16, F18, F19, Z71.5, Z72.2 |
| Fluid And Electrolyte Disorders | 11 | E22.2, E86, E87 |
| Hypertension (combined uncomplicated and complicated) | -1 | I10, I11, I12, I13, I15 |
| Hypothyroidism | 0 | E00, E01, E02, E03, E89.0 |
| Liver Disease | 4 | B18, I85, I86.4, I98.2, K70, K71.1, K71.3, K71.4, K71.5, K71.7, K72, K73, K74, K76.0, K76.2, K76.9, Z94.4 |
| Lymphoma | 6 | C81, C82, C83, C84, C85, C88, C96, C90.0, C90.2 |
| Metastatic Cancer | 14 | C77, C78, C79, C80 |
| Neurological Disorders, other | 5 | G10, G11, G12, G13. G20, G21, G22, G25.4, G25.5, G31.2, G31.8, G31.9, G32, G35, G36, G37, G40, G41, G93.1, G93.4, R47.0, R56 |
| Obesity | -5 | E66 |
| Paralysis | 5 | G04.1, G11.4, G80.1, G80.2, G81, G82, G83.0, G83.1, G83.2, G83.3, G83.4, G83.9 |
| Peptic Ulcer Disease, Excluding Bleeding | 0 | K25.7, K25.9, K26.7, K26.9, K27.7, K27.9, K28.7, K28.9 |
| Peripheral Vascular Disorders | 3 | I70, I71, I73.1, I73.8, I73.9, I77.1, I79.0, I79.2, Z95.81, Z95.88, Z95.9 |
| Psychoses | -5 | F20, F22, F23, F24, F25, F28, F29, F30.2, F31.2, F31.5 |
| Pulmonary Circulation Disorders | 6 | I26, I27, I28.0, I28.8, I28.9 |
| Rheumatoid Arthritis / Collagen Vascular Diseases | 0 | L94.0, L94.1, L94.3, M05, M06, M08, M12.0, M12.3, M30, M31.0, M31.1, M31.2, M31.3, M32, M33, M34, M35, M45, M46.1, M46.8, M46.9 |
| Solid Tumor Without Metastases | 7 | C00, C01, C02, C03, C04, C05, C06, C07, C08, C09, C10, C11, C12, C13, C14, C15, C16, C17, C18, C19, C20, C21, C22, C23, C24, C25, C26, C30, C31, C32, C33, C34, C37, C38, C39, C40, C41, C43, C45, C46, C47, C48, C49, C50, C51, C52, C53, C54, C55, C56, C57, C58, C60, C61, C62, C63, C64, C65, C66, C67, C68, C69, C70, C71, C72, C73, C74, C75, C76, C97 |
| Valvular Heart Disease | 0 | I05, I06, I07, I08, I09.1, I34, I35, I36, I37, I38, I39, Q23.0, Q23.1, Q23.2, Q23.3, Z95.2, Z95.3, Z95.4 |
| Weight Loss | 9 | E40, E41, E42, E43, E44, E45, E46, R63.4, R64 |

ICD-10-GM = German Modification of the International Statistical Classification of Diseases and Related Health Problems Version 10

**Table S2: Baseline characteristics total dataset, training and testing cohort**

| Variable | Total | Training cohort | Testing cohort | P-Value |
| --- | --- | --- | --- | --- |
| N (SARI cases) | 241,988 | 181,574 | 60,414 |  |
| Age |  |  |  |  |
| <65 | 16.2% (39186/241988) | 16.2% (29406/181574) | 16.2% (9780/60414) | 0.999 |
| 65-74 | 49% (118515/241988) | 49% (88916/181574) | 49% (29599/60414) | 0.995 |
| ≥75 | 34.8% (84287/241988) | 34.8% (63252/181574) | 34.8% (21035/60414) | 0.997 |
| Gender |  |  |  |  |
| Female | 43.8% (106004/241988) | 44% (79806/181574) | 43.4% (26198/60414) |  |
| Male | 56.2% (135984/241988) | 56% (101768/181574) | 56.6% (34216/60414) | 0.041 |
| ICU treatment | 14.7% (35610/241988) | 14.7% (26690/181574) | 14.8% (8920/60414) | 0.925 |
| Hospital-acquired SARI | 20% (48473/241988) | 19.9% (36223/181574) | 20.3% (12250/60414) | 0.220 |
| Influenza | 4.2% (10238/241988) | 4.2% (7669/181574) | 4.3% (2569/60414) | 0.955 |
| Viral pneumonia other than influenza | 1.5% (3632/241988) | 1.5% (2715/181574) | 1.5% (917/60414) | 0.925 |
| Bacterial pneumonia | 15.6% (37713/241988) | 15.6% (28317/181574) | 15.6% (9396/60414) | 0.969 |
| Other pneumonia | 56.6% (136988/241988) | 56.4% (102480/181574) | 57.1% (34508/60414) | 0.014 |
| Other lower respiratory tract infections | 24.3% (58840/241988) | 24.4% (44346/181574) | 24% (14494/60414) | 0.100 |
| Congestive heart failure | 33.8% (81886/241988) | 33.8% (61443/181574) | 33.8% (20443/60414) | 1.000 |
| Cardiac arrhythmias | 31% (75126/241988) | 31.1% (56514/181574) | 30.8% (18612/60414) | 0.345 |
| Valvular disease | 11.8% (28512/241988) | 11.8% (21492/181574) | 11.6% (7020/60414) | 0.359 |
| Pulmonary circulation disorders | 8.1% (19652/241988) | 8.1% (14683/181574) | 8.2% (4969/60414) | 0.559 |
| Peripheral vascular disorders | 9.9% (24000/241988) | 9.9% (18032/181574) | 9.9% (5968/60414) | 0.933 |
| Hypertension, uncomplicated | 35.9% (86769/241988) | 35.9% (65096/181574) | 35.9% (21673/60414) | 0.995 |
| Hypertension, complicated | 16.1% (38874/241988) | 16.1% (29185/181574) | 16% (9689/60414) | 0.979 |
| Paralysis | 6.5% (15709/241988) | 6.4% (11634/181574) | 6.7% (4075/60414) | 0.014 |
| Other neurological disorders | 10.7% (25905/241988) | 10.6% (19329/181574) | 10.9% (6576/60414) | 0.256 |
| Chronic pulmonary disease | 19% (45891/241988) | 18.9% (34347/181574) | 19.1% (11544/60414) | 0.581 |
| Diabetes, uncomplicated | 13.4% (32492/241988) | 13.5% (24531/181574) | 13.2% (7961/60414) | 0.115 |
| Diabetes, complicated | 11.9% (28903/241988) | 11.9% (21636/181574) | 12% (7267/60414) | 0.760 |
| Hypothyroidism | 9.8% (23643/241988) | 9.7% (17651/181574) | 9.9% (5992/60414) | 0.368 |
| Renal failure | 34.8% (84217/241988) | 34.8% (63150/181574) | 34.9% (21067/60414) | 0.919 |
| Liver disease | 5.1% (12390/241988) | 5.1% (9308/181574) | 5.1% (3082/60414) | 0.972 |
| Metastatic cancer | 5.1% (12454/241988) | 5.1% (9270/181574) | 5.3% (3184/60414) | 0.283 |
| Solid tumor without metastasis | 9.1% (22057/241988) | 9.1% (16481/181574) | 9.2% (5576/60414) | 0.527 |
| Coagulopathy | 6.1% (14663/241988) | 6% (10922/181574) | 6.2% (3741/60414) | 0.287 |
| Obesity | 11.2% (27139/241988) | 11.2% (20379/181574) | 11.2% (6760/60414) | 0.974 |
| Weight Loss | 14% (33947/241988) | 14.1% (25578/181574) | 13.9% (8369/60414) | 0.357 |
| Fluid and electrolyte disorders | 43.1% (104383/241988) | 43.1% (78273/181574) | 43.2% (26110/60414) | 0.893 |
| Depression | 5.5% (13209/241988) | 5.5% (10075/181574) | 5.2% (3134/60414) | 0.003 |

ICU = Intensive care unit; SARI = severe acute respiratory infection

**Table S3: DeLong’s test for pairwise comparison of ROC AUCs**

|  | RF | NNET | XGBoost |
| --- | --- | --- | --- |
| GLM | 0.291 | <0.001 | <0.001 |
| RF | // | <0.001 | <0.001 |
| NNET | // | // | 0.514 |

ROC AUCs are compared pairwise using the DeLong’s test (DeLong et al., 1988). P-values are adjusted for multiple comparisons using the Holm’s method (Holm et al., 1979). AUC = area under the curve; GLM = generalized linear models; NNET = single layer neural network; RF = random forest; ROC = receiver operating characteristic; XGBoost = extreme gradient boosting

**Table S4: Performance metrics (model testing)**

| Algorithm | Precision | Recall | F1-score | NPV |
| --- | --- | --- | --- | --- |
| GLM | 0.329 | 0.588 | 0.422 | 0.940 |
| RF | 0.340 | 0.560 | 0.423 | 0.937 |
| NNET | 0.323 | 0.614 | 0.423 | 0.943 |
| XGBoost | 0.347 | 0.548 | 0.425 | 0.936 |

GLM = generalized linear models; NNET = single layer neural network; NPV = Negative predictive value; RF = random forest; ROC = receiver operating characteristic; XGBoost = extreme gradient boosting

**Figure S1 (A-D): SHAP (SHapley Additive exPlanations) analysis for variable importance**

**
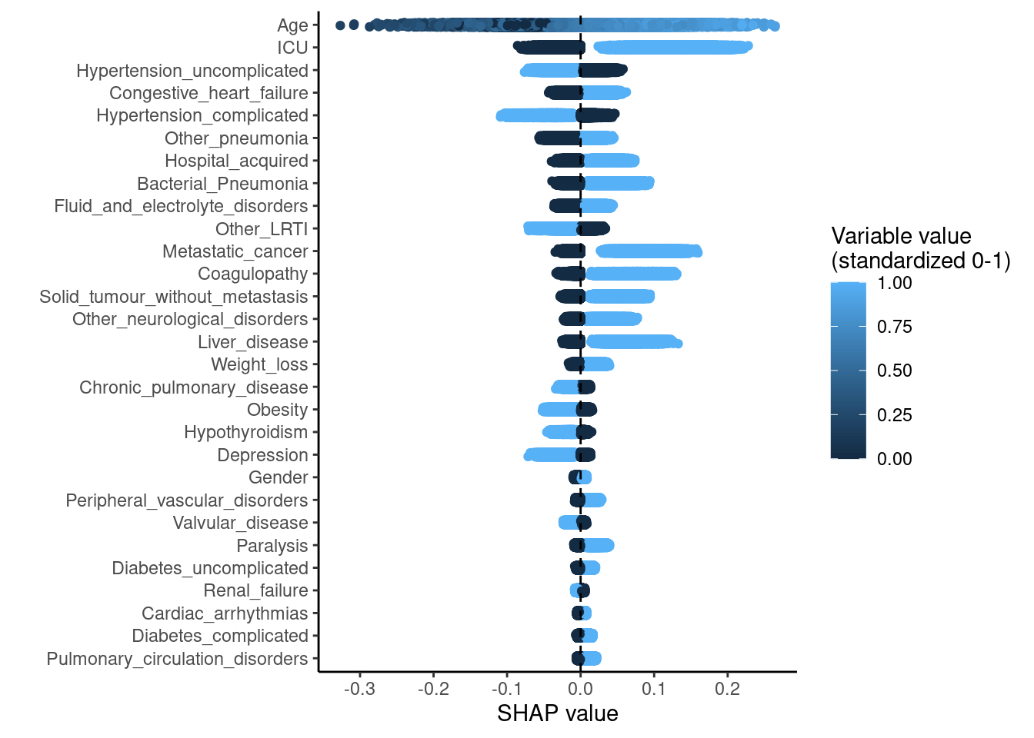
**

A: SHAP summary plot for GLM (generalized linear models). Variables are ordered by importance (top to bottom). For more information, please see: *Lundberg SM, Lee S-I: A unified approach to interpreting model predictions. Advances in neural information processing systems 2017.*

**
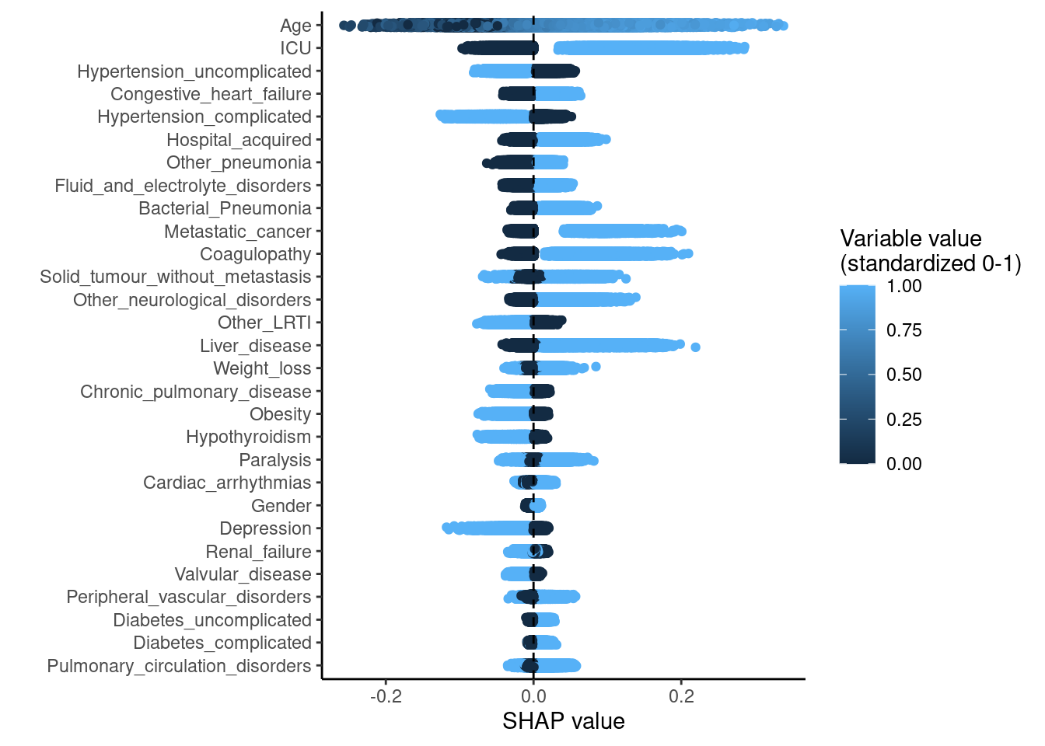
**

B: SHAP summary plot for NNET (single layer neural network). Variables are ordered by importance (top to bottom). For more information, please see: *Lundberg SM, Lee S-I: A unified approach to interpreting model predictions. Advances in neural information processing systems 2017.*

**
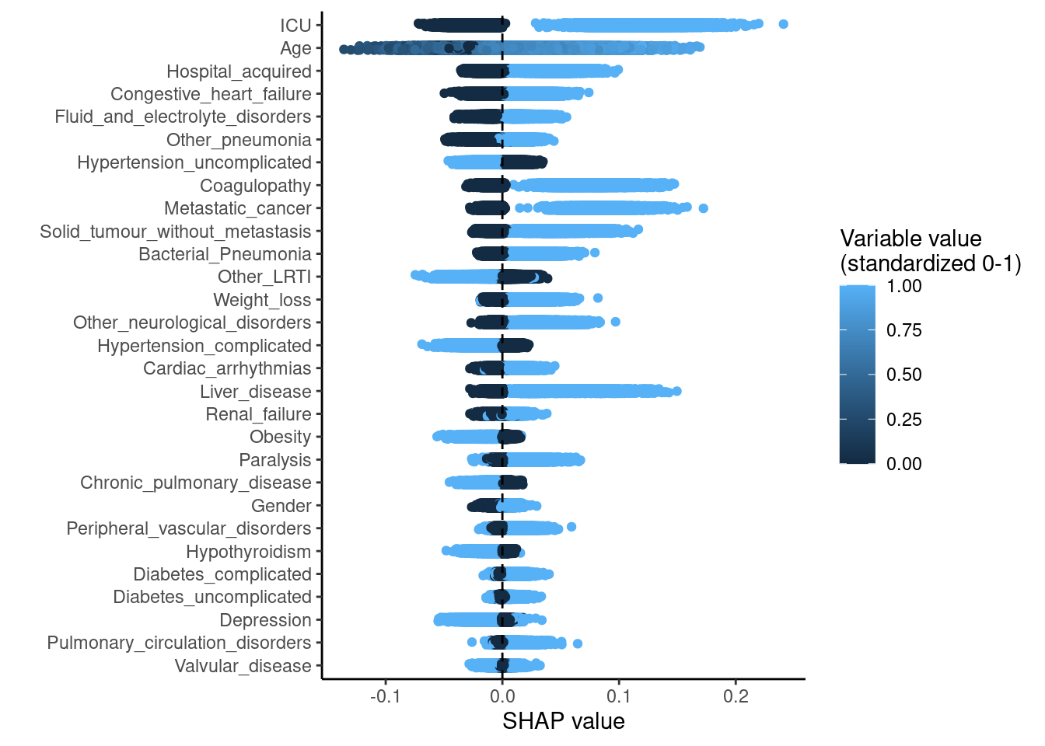
**

C: SHAP summary plot for RF (random forest). Variables are ordered by importance (top to bottom). For more information, please see: *Lundberg SM, Lee S-I: A unified approach to interpreting model predictions. Advances in neural information processing systems 2017.*

**
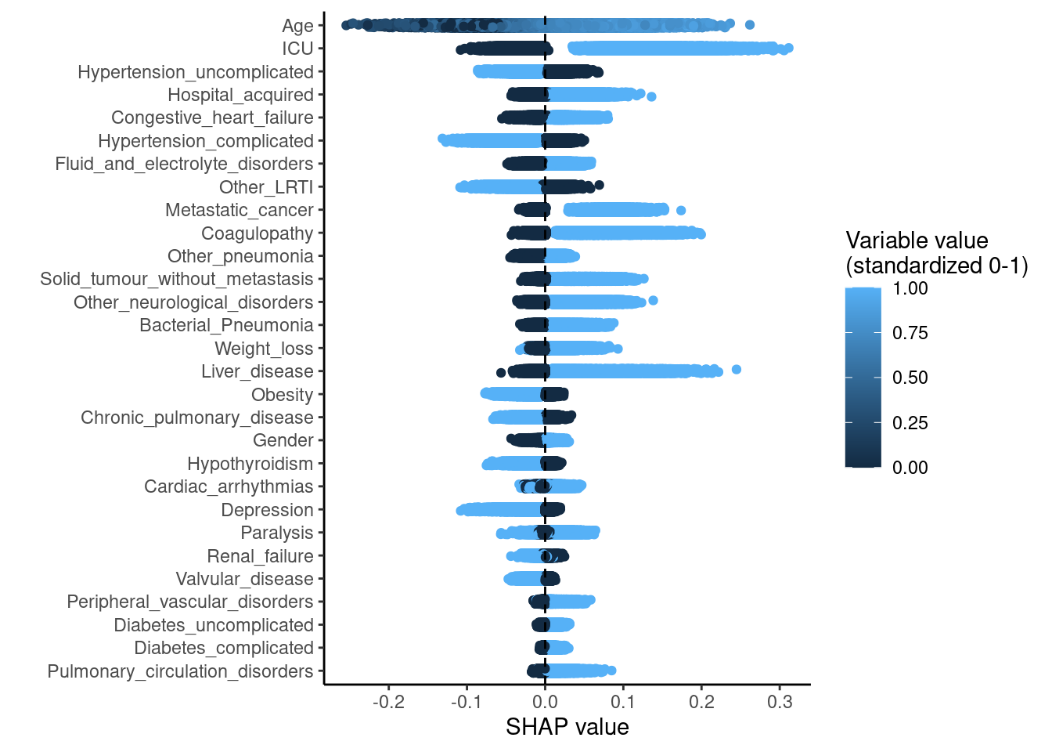
**

D: SHAP summary plot for XGBoost (extreme gradient boosting). Variables are ordered by importance (top to bottom). For more information, please see: *Lundberg SM, Lee S-I: A unified approach to interpreting model predictions. Advances in neural information processing systems 2017.*
